# Exosomes: Innocent Bystanders or Critical Culprits in Neurodegenerative Diseases

**DOI:** 10.3389/fcell.2021.635104

**Published:** 2021-05-13

**Authors:** Margarida Beatriz, Rita Vilaça, Carla Lopes

**Affiliations:** ^1^CNC-Center for Neuroscience and Cell Biology, University of Coimbra, Coimbra, Portugal; ^2^IIIUC-Institute for Interdisciplinary Research, University of Coimbra, Coimbra, Portugal

**Keywords:** exosomes, central nervous system, neurodegenerative diseases, biomarkers, neural-derived exosomes

## Abstract

Extracellular vesicles (EVs) are nano-sized membrane-enclosed particles released by cells that participate in intercellular communication through the transfer of biologic material. EVs include exosomes that are small vesicles that were initially associated with the disposal of cellular garbage; however, recent findings point toward a function as natural carriers of a wide variety of genetic material and proteins. Indeed, exosomes are vesicle mediators of intercellular communication and maintenance of cellular homeostasis. The role of exosomes in health and age-associated diseases is far from being understood, but recent evidence implicates exosomes as causative players in the spread of neurodegenerative diseases. Cells from the central nervous system (CNS) use exosomes as a strategy not only to eliminate membranes, toxic proteins, and RNA species but also to mediate short and long cell-to-cell communication as carriers of important messengers and signals. The accumulation of protein aggregates is a common pathological hallmark in many neurodegenerative diseases, including Alzheimer’s disease, Parkinson’s disease, Huntington’s disease, amyotrophic lateral sclerosis, and prion diseases. Protein aggregates can be removed and delivered to degradation by the endo-lysosomal pathway or can be incorporated in multivesicular bodies (MVBs) that are further released to the extracellular space as exosomes. Because exosome transport damaged cellular material, this eventually contributes to the spread of pathological misfolded proteins within the brain, thus promoting the neurodegeneration process. In this review, we focus on the role of exosomes in CNS homeostasis, their possible contribution to the development of neurodegenerative diseases, the usefulness of exosome cargo as biomarkers of disease, and the potential benefits of plasma circulating CNS-derived exosomes.

## Introduction

**FIGURE 1:**
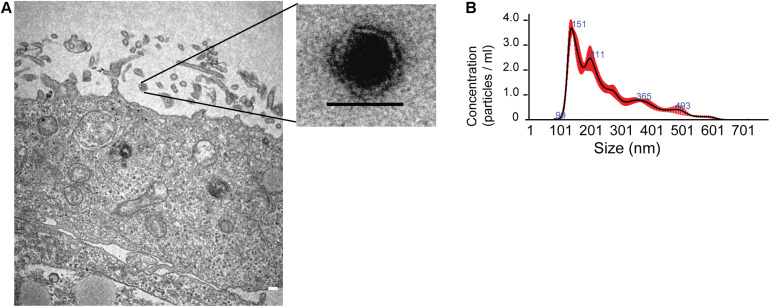
Characterization of exosomes released from human neural stem cells differentiated from Huntington’s Disease iPS cells. **(A)** Left: Electron micrographs of exosomes released from neural stem cell membrane, scale 200 nm. Right: Exosome with a lipid bilayer membrane sizing 50 nm. **(B)** Nanoparticle Tracking Analysis (NTA) of exosomes with sizes ranging from 67.7 to 152.1 nm (10th; 90th percentile) cited from Lopes et al. (2020).

Extracellular vesicles (EVs) are secreted by all types of cells, including neuronal cells, and can be found in almost all body fluids, including urine, saliva, blood, and cerebrospinal fluid (CSF) (Faure et al., 2006; Yanez-Mo et al., 2015). EVs can be divided according to their size and mechanism of release. In 2018, the International Society for Extracellular Vesicles (ISEV) endorsed the use of a new standardized nomenclature considering terms for subtypes that refer to physical properties such as size (small < 200 nm) or biochemical composition, among others (Théry et al., 2018). Apoptotic bodies and microvesicles (>100 nm in diameter) are released directly from the outward budding of the plasma membrane, while exosomes (30–150 nm in diameter, most common size described in the literature) are released after fusion of endosome-derived vesicles with the plasma membrane (Figure 1). Thus, different pools of EVs are characterized based on distinct intracellular origins and biogenesis (Kowal et al., 2014). Exosome biogenesis and secretion are modulated by different cellular stress and/or pathological conditions (Raposo and Stoorvogel, 2013; Record et al., 2014; Becker et al., 2016). These vesicles act as selective transporters of proteins, lipids, and genetic material, thus constituting an important intercellular communication system, capable of influencing distinct cellular activities in the recipient cells (Mathivanan et al., 2010; Yanez-Mo et al., 2015). The growing interest in EVs has been reflected in the creation of distinct databases that compile data on exosome content such as Exocarta (Mathivanan and Simpson, 2009), miRandola (Russo et al., 2018), EVpedia (Kim et al., 2013), and Vesiclepedia (Kalra et al., 2012), which are constantly updated with released studies. For the scope of this review, we will focus on exosomes and their biological function in central nervous system (CNS) homeostasis and discuss the role, as biomarkers, in the context of neurological disorders, particularly CNS-derived exosomes that can be isolated from the blood.

### Exosome Biogenesis and Cargo Loading

Exosomes originate during the maturation of early endosomes through the inward budding of the endosomal membrane that generates intraluminal vesicles (ILVs), resulting in the formation of late endosomes (LEs) with multiple small vesicles termed MVB (Huotari and Helenius, 2011). During this process, cytosolic portions, proteins, and nucleic acids are enveloped into the vesicles, and the resulting MVBs can be either targeted to lysosomal degradation or fused with the plasma membrane, releasing the ILVs to the extracellular space as exosomes.

Exosome biogenesis can occur through an Endosomal Sorting Complex Required for Transport (ESCRT)-dependent mechanism or by an ESCRT-independent pathway. The first relies on multiple proteins that assemble into four ESCRT complexes (ESCRT-0, I, II, and III) and accessory proteins, such as an AAA-ATPase, VPS4 complex, and ALIX that are involved in MVB budding and loading (Babst et al., 2002; Henne et al., 2011; Colombo et al., 2014).

The exosome biogenesis also occurs through an ESCRT-independent pathway mediated by tetraspanins and ceramide-enriched lipid rafts (Trajkovic et al., 2008; Van Niel et al., 2011). Tetraspanins are recruited at early steps to endosome membranes, before ILV formation (Escola et al., 1998; Pols and Klumperman, 2009) and at least CD63, CD9, CD81, and CD82 are found in endosome and exosome membranes (Andreu and Yanez-Mo, 2014).

**FIGURE 2:**
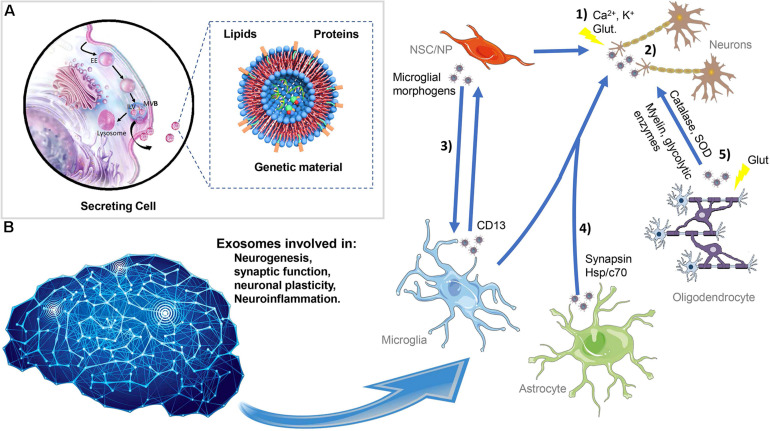
Exosome biogenesis, secretion, and interaction in the central nervous system. **(A)** Exosomes originated in the endocytic pathway as ILVs resulting in the formation of LE with multiple small vesicles termed MVBs. During this process, exosomes are loaded with proteins, lipids, and genetic material. Afterward, the MVBs can be destined for lysosomal degradation or fused with the plasma membrane and the exosomes released into the extracellular space. The exosomes have a complex composition of proteins such as ALIX, TSG101 and flotillin, Rab family, tetraspanins (e.g., CD63, CD81, and CD9), mitochondrial proteins, and others; lipids including cholesterol, sphingomyelin, glycosphingolipids, and ceramide; and nucleic acids especially RNA and also nuclear and mitochondrial DNA. **(B)** Exosomes can be released from NSC/NP, neurons, astrocytes, oligodendrocytes, and microglia acting in proximal or distal cells. Exosomes can be shuttled between the different neural cells modulating distinct neuronal processes such as neurogenesis, synaptic function, neuronal plasticity, and neuroinflammation. (1) In neurons, exosomes are released in response to stimulus, such as calcium influx, glutamatergic synaptic activity, and potassium-induced depolarization. (2) The exosome signaling pathways include neuron-to-neuron communication. (3) NSC can influence the microglia function, and exosomes released from glial cells exert effects on NSC. (4) Additionally, exosomes originated in astrocytes can be fundamental for the trophic support of neurons. (5) Oligodendrocytes can also shuttle to neuron exosomes containing myelin proteins and glycolytic enzymes. ILVs, intraluminal vesicles; EEs, early endosomes; MVBs, multivesicular bodies, NSC/NP, neural stem/progenitor cells.

Ceramide and its derived metabolites are organized in raft-based microdomains that interact with proteins, such as flotillins. These lipid-enriched structures are involved not only in endosomal membrane invagination for ILV formation (Trajkovic et al., 2008) but also in cargo loading. The selective cargo loading occurs during exosome biogenesis through tetraspanin-dependent and/or ESCRT-dependent mechanisms. The composition and quantity of exosome released are dictated by specific molecules present in the membrane and lumen of ILVs and in the membrane of MVB, which is determinant for their selective trafficking toward plasma membrane for exosome release (Figure 2A).

#### Proteins

Proteins that are commonly found in exosomes are the ones involved in their biogenesis and are located in the endosomal membrane. The proteins detected in exosome fractions comprise components of the ESCRT machinery such as ALIX, TSG101, and flotillins (Kowal et al., 2016), proteins responsible for transport and fusion of MVB with the plasma membrane (RAB27A, RAB11B) and tetraspanins (e.g., CD63, CD81, and CD9) (Ostrowski et al., 2010; Kowal et al., 2016). Proteins associated with lipid rafts are found in exosomes, as they originate primarily from early endosomes formed by invagination of the plasma membrane (de Gassart et al., 2003). Membrane-associated and signaling proteins were found in exosomes released from stem cells after stimulation of their trafficking to lipid rafts, thus supporting a raft-mediated protein sorting mechanism (Lin et al., 2017).

Ubiquitinated proteins were detected in exosomes (Buschow et al., 2005) and might be sorted into ILVs by an ESCRT-dependent mechanism (MacDonald et al., 2012). ESCRT-0 can recognize and bind ubiquitin-like molecules, such as small ubiquitin-like modifiers (SUMO), thus recruiting SUMOylated proteins like RNA-binding proteins (RBPs) (Villarroya-Beltri et al., 2013). NEDD4, an E3 ubiquitin-protein ligase that recognizes PPXY motifs in cargo proteins, might also be involved in exosome loading with ubiquitinated proteins (Putz et al., 2008). Recently, it has been described that about 10% of exosome protein content are mitochondria-associated proteins (Choi et al., 2013). Despite the fact that initial studies have shown that mitochondrial proteins are absent in tumor-derived exosomes (Mears et al., 2004), further developments of the methodologies for exosome proteomic analysis revealed the presence of mitochondrial proteins in exosomes (Burke et al., 2014; Wang et al., 2020). A recent study showed that exosomes released from Huntington’s disease (HD) patient-derived iPSC also carried mitochondrial proteins (Lopes et al., 2020). A preliminary report also identified the presence of functional mitochondria in neural stem cell-derived exosomes (Peruzzotti-Jametti et al., 2020). The generation of mitochondrial-derived vesicles (MDVs) containing malfunctioning parts (proteins or peptides) seems to constitute a novel mitochondrial quality control mechanism. The MDVs can enter the secretory pathway by fusing with LE/MVB for further degradation into lysosomes (Soubannier et al., 2012), but, alternatively, they can be sorted to the plasma membrane, thus releasing the mitochondria content through exosomes.

Most of the proteins used as exosome markers are found in exosomes from different sources and are not exclusive to neuronal cells. Despite the enrichment of exosomes with some of these proteins, they can also be found in other types of EVs, as they are membrane-associated proteins (Lötvall et al., 2014). This raises concerns on the definition of exosome-specific cargo and on the characterization of cell-specific exosome fractions. Still, some cell-specific proteins can be used to distinguish the source of exosomes from different neuronal cells. For instance, neural secreted exosomes present cell adhesion molecule L1 (L1CAM) and glutamate receptors (Faure et al., 2006; Lachenal et al., 2011), oligodendrocyte exosomes contain specific myelin proteins (Kramer-Albers et al., 2007), and microglial exosomes contain CD13 and monocarboxylate transporter 1, which are cell specific (Potolicchio et al., 2005). Therefore, it is expected that exosomes might have a specific signature that correlates with their cellular origin and further research will bring light on their physiological roles.

#### Lipids

Lipids in exosomes share similarities with plasma membrane composition, particularly lipids that are normally found in lipid rafts such as plasma membrane composition cholesterol, sphingomyelin, glycosphingolipids, and ceramide (de Gassart et al., 2003; Llorente et al., 2013; Skotland et al., 2017). The raft-like regions are important platforms for protein stabilization in the outer left of the ILV: cholesterol is important for the assembly of ESCRT protein complexes (Boura et al., 2012) while lysophosphatidic acid (LBPA), a glycerophospholipid mainly found in MVB, stabilizes ALIX (Kufareva et al., 2014). Modulating the lipid content of exosomes affects their release. Treatment of oligodendroglial cells with an inhibitor of cholesterol egress from lysosomes (U18666A) or with a soluble cholesterol analog compromises exosome release (Strauss et al., 2010). On the other hand, cholesterol depletion induced by methyl-β-cyclodextrin treatment led to an increase of exosome release (Llorente et al., 2007). Exosomes are uniquely enriched in phosphatidylserine in the outer face of the membrane, which may help in the internalization process by the recipient cells (Morelli et al., 2004). Also, ceramide and glycosphingolipids are found in exosomes as they are involved in their biogenesis and release. Inhibition of neutral sphingomyelinase 2 (nSMase2), an enzyme that metabolizes sphingomyelin into ceramide, altered the loading of CD63, flotillin, and proteolipid proteins into exosomes and also impaired exosome secretion (Trajkovic et al., 2008; Yuyama et al., 2012; Menck et al., 2017; Miranda et al., 2018). Inhibition of ceramide conversion into sphingomyelin by siRNA or chemical inhibition of sphingomyelin synthase 2 (SMS2) promotes exosome secretion (Yuyama et al., 2012; Dinkins et al., 2014).

#### Genetic Material

Exosomes are natural carriers of genetic material, with almost any type of RNA being found inside, and to some extent also nuclear and mitochondrial DNA (mtDNA) (Valadi et al., 2007; Waldenstrom et al., 2012; Ridder et al., 2014). Exosomes are particularly enriched in small RNAs around 200 nucleotides in length, and only a few have more than 3 Kb, which supports the notion that most of the mRNA and long non-coding RNA detected are fragmented (Crescitelli et al., 2013). RNA loading into ILVs is mediated by RBPs that assist in the transport of RNAs to raft-like regions by a selective mechanism that involves specific RNA sequences and RNA hydrophobic modifications (Janas et al., 2004; Batagov et al., 2011).

One of the most important exosome cargoes are miRNAs, which are non-coding RNAs with 17–21 nucleotides, that regulate protein expression by binding to the untranslated regions (UTR) of mRNAs thus inhibiting their translation. Several studies have shown enrichment of selective miRNAs in exosomes, which suggests a selective mechanism of miRNA loading during the vesicle biogenesis (Guduric-Fuchs et al., 2012).

The packaging of miRNAs into MVBs is favored by the presence of specific exosome-sorting motifs in the 3′ portion of miRNA that is recognized by RBPs such as the chaperone hnRNPA2B1 (Heterogeneous Nuclear Ribonucleoprotein A2/B1), by a ceramide-dependent mechanism (Villarroya-Beltri et al., 2013). Also, 3′end uridylation of miRNA is involved with its sorting into EVs, demonstrating that posttranslational modifications regulate selective sorting of miRNA (Wyman et al., 2011; Koppers-Lalic et al., 2014). Mature miRNAs can interact with proteins forming the miRNA-induced silencing complex (miRISC). Argonaute 2 (Ago2) is a component of miRISC that localizes in MVBs, thus promoting miRNA loading (Gibbings et al., 2009; Guduric-Fuchs et al., 2012; Cha et al., 2015). Some studies have shown that Ago2 is present in exosomes (Goldie et al., 2014) while others reported its localization only at MVBs inside cells (Gibbings et al., 2009). More recent studies showed that the impairment of Ago2 localization in MVB affected its secretion in exosomes (McKenzie et al., 2016). In agreement, knockout of Ago2 impaired the export of selective miRNAs in HEK293T-derived exosomes (Guduric-Fuchs et al., 2012).

The miRNAs are also players involved in the loading mechanism of mRNA into exosomes. Specific sequences in 3′UTR of mRNA, with approximately 25 nucleotides and a CTGCC motif within a stem-loop structure, act as a sorting sequence to exosomes through a mechanism mediated by miR-1289 (Bolukbasi et al., 2012).

Exosomes also contain mtDNA (Guescini et al., 2010; Sansone et al., 2017) and single- and double-stranded DNA (Balaj et al., 2011; Yang et al., 2017). The secretion of DNA by exosomes may constitute an alternative process to maintain intracellular homeostasis by eliminating harmful cytoplasmic DNA (Takahashi et al., 2017). Moreover, part of the exosome DNA was bound to histones, which have been profiled in distinct exosome proteomic analysis (Simpson et al., 2008). So, the authors speculate that DNA loading into exosomes might depend on histones (Takahashi et al., 2017).

In a recent report, the authors employed a novel approach for exosome isolation with a combined methodology of high-resolution density gradient fractionation followed by a direct immunoaffinity capture to selectively isolate exosomes (Jeppesen et al., 2019). The study showed contradicting results, such as the presence of miRNA in non-vesicular fractions rather than in exosome fractions. This was supported by the absence of Ago2 or any miRNA machinery and RBPs in exosomes, in contrast with previous studies (Melo et al., 2014). Also, this study showed that isolated exosomes lacked dsDNA. Instead, this molecule is co-purified with these vesicles when standard isolation protocols are used. The authors proposed an EV-independent mechanism for dsDNA extracellular release, contradicting previous findings (Thakur et al., 2014). Most of the initial studies reporting dsDNA within exosomes were performed in cell conditional media, which might lead to premature conclusions on exosomes circulating on body fluids without validated protocols and standardized nomenclature (Ludwig et al., 2019). The Jeppesen study among others has pointed out that the isolation and purification methods have an important outcome in the final exosome content characterization and it is urgent to establish unified criteria for vesicle standard definition (size and density) and characterization of their cargo (Théry et al., 2018; Jeppesen et al., 2019; Witwer and Thery, 2019). Researchers have to be mindful of setting a subset characterization of exosome fractions before taking general conclusions from distinct studies. The vast majority of the current knowledge on the exosome field was obtained from cell line studies using conditional media. This remains the best approach for examining exosome content derived from a single or well-defined cell-type population and their function on other cells (Abramowicz et al., 2018).

### The Transport of MVB to the Plasma Membrane and Exosome Release

The interaction of MVBs with actin and microtubules is essential for their transport to the plasma membrane (Villarroya-Beltri et al., 2014). The translocation of MVB toward the plasma membrane depends on several molecules via the cytoskeleton (Mittelbrunn et al., 2015). Kifc2 is a neuronal protein that moves directionally toward the plus end of microtubules and was found to be associated with MVB, suggesting a possible role in MVB transport to the neuronal plasma membrane (Saito et al., 1997). Rab GTPases such as RAB11, RAB27A/B, and RAB35 are mediators of selective sorting of MVB to the plasma membrane and exosome release (Hsu et al., 2010; Ostrowski et al., 2010; Escudero et al., 2014). RAB11 and p75 neurotrophin receptors are involved in directing MBV to the plasma membrane and exosome release in neuronal cells (Escudero et al., 2014). Also, Rab35 is involved in the mechanism of exosome release by oligodendrocytes (Fruhbeis et al., 2013). The MVBs are decorated with tethering protein complexes, such as HOPS and SNAREs, that mediate the fusion of these vesicles with the plasma membrane (Itakura et al., 2012; Jiang et al., 2014). The presence of tetraspanins (e.g., CD63) and lysosomal-associated membrane proteins LAMP1 and LAMP2 in late endosomes also facilitate the fusion of MVB with the plasma membrane (Jaiswal et al., 2002).

### Uptake of Exosomes by Recipient Cells

After secretion, the exosomes will dock into the membrane of the target cells and activate signaling events or will be internalized through specific receptor–ligand interactions (Mulcahy et al., 2014). The transmembrane proteins present in the surface of exosomes (tetraspanins) can be recognized by signaling receptors in the target cells (Munich et al., 2012), resulting in activation of transducing pathways and modulation of intracellular processes without entering the target cell. Exosomes can merge with the plasma membrane of target cells releasing its cargo directly into the cytosol by a low pH-dependent mechanism (Parolini et al., 2009). However, the main route for exosome uptake seems to be mediated by endocytic events. Exosome uptake can occur by clathrin-mediated (Escrevente et al., 2011) or caveolin-dependent endocytosis (Nanbo et al., 2013), and the presence of lipid rafts in the membrane facilitates the process (Izquierdo-Useros et al., 2009; Svensson et al., 2013). After internalization, exosomes are sorted into LE/MVB with two possible fates: to be released again to neighboring cells or to be degraded after fusion of LE/MVB with lysosomes (Tian et al., 2013).

The uptake of exosomes by brain cells seems to be cell-type dependent. For instance, neurons and glial cells seem to uptake exosomes by clathrin-mediated endocytosis (Alvarez-Erviti et al., 2011b; Fruhbeis et al., 2013). Some neurons can also use specific receptors from the SNARE family, such as SNAP25, for exosome uptake (Zhang et al., 2017a). Curiously, the uptake of exosomes seems to be a selective pathway; exosomes derived from cortical neurons were primarily internalized by hippocampal neurons whereas exosomes released by neuroblastoma cell line N2A were taken up by astrocytes and oligodendrocytes (Chivet et al., 2014). Also, exosomes derived from oligodendrocytes were mainly internalized by microglia but not by neurons or astrocytes (Fitzner et al., 2011). Thus, the mechanisms that regulate the selective targeting of exosomes to recipient cells can determine their biological effect. Moreover, the uptake of exosomes was also more active in pre-synaptic regions, which might indicate that these vesicles use constitutive endocytosis processes at these regions for neuronal cell entrance (Granseth et al., 2006; Chivet et al., 2014). Nevertheless, the exact mechanism involved in exosome entrance into neuronal cells is still uncharacterized.

## Exosomes and the CNS

If originally exosomes were considered to be disposable vehicles for the elimination of cellular components, now multiple studies demonstrated that exosomes play multiple physiological roles in the nervous system. Exosomes can be released from all brain cells, including neural stem/progenitor cells (Ma et al., 2019), neurons (Faure et al., 2006), astrocytes (Taylor et al., 2007; Fitzner et al., 2011), and microglia (Xia et al., 2019) acting in proximal or distal cells. Importantly, exosomes can be shuttled between the different neural cells modulating distinct neuronal processes such as neurogenesis (Luarte et al., 2017), synaptic function (Lachenal et al., 2011), neuronal plasticity (Lafourcade et al., 2016), and neuroinflammation (Dalvi et al., 2017; Figure 2B).

Neural stem/neural progenitor cells (NSCs/NPCs), found in neurogenic niches, can secrete or uptake exosomes, thus playing key roles in neuronal development (Ma et al., 2019). Exosomes derived from neural stem cells were able to promote neuronal growth and differentiation as their protein cargo affects neuronal development and function (Sharma et al., 2019). The exosomes derived from NSCs carry specific miRNA that targets neural regeneration, neuroprotection, and neural plasticity (Stevanato et al., 2016). Moreover, miRNA present in exosomes released from NSCs at the hypothalamic subventricular zone might play a beneficial role in the control of aging (Zhang et al., 2017b).

Exosomes are also important mediators of neuron-to-neuron communication. The secreted exosomes enter the recipient cells and can be re-secreted along with the recipient neuron endogenously formed exosomes, which facilitates specialized long-distance communication within a cell type (Polanco et al., 2018). Neuronal exosomes are mostly released from a somato-dendritic compartment in response to calcium influx and glutamatergic synaptic activity (Lachenal et al., 2011; Men et al., 2019). Moreover, potassium-induced depolarization stimulates the release of exosomes at synaptic terminals containing subunits of glutamate receptors (AMPA), GPI-anchored prion proteins, and L1 cell adhesion molecule (L1CAM) (Faure et al., 2006). The presence of proteins involved in neuronal function, such as amyloid precursor protein (APP) (Vingtdeux et al., 2007) or adaptor protein Ndfip1 (Putz et al., 2008) in exosomes further supported their modulatory role in synaptic function. Neuronal-derived exosomes (NDEs) containing microtubule-associated protein 1B (MAP1B) and a specific subset of miRNA related to synaptic plasticity were also described (Goldie et al., 2014). The release of exosomes by neurons is an important event in neuron-to-neuron communication. The Synaptotagmin 4 (Syt4)-containing exosomes were implicated in retrograde postsynaptic signaling by mediating activity-dependent presynaptic growth and neurotransmitter release (Korkut et al., 2013). Neuronal exosomes also carry Wnt proteins thus modulating the Wnt signaling pathway in the recipient cells (Korkut et al., 2009). Exosomes secreted by neurons contain a subset of miRNA that is distinct from the donor cells (Men et al., 2019), which support a selective packaging mechanism.

Trophic support of neurons is assisted by exosomes released from oligodendrocytes and astrocytes (Fruhbeis et al., 2012). Glutamate release by neurons triggers the secretion of exosomes enriched in specific proteins, and RNA from oligodendrocytes was shown to improve the cellular viability of neuronal cells under stress conditions (Fruhbeis et al., 2013). Moreover, the presence of catalase and superoxide dismutase in oligodendroglial exosomes might help neuronal survival under stress conditions of oxygen/glucose deprivation (Frohlich et al., 2014). Exosomes can mediate communication between oligodendrocytes and axons by supplying neurons with myelin proteins and glycolytic enzymes and substrates (Kramer-Albers et al., 2007; Kramer-Albers and White, 2011). During CNS tissue regeneration, oligodendrocytes communicate with microglia via exosomes to induce microglia-mediated elimination of oligodendroglial membranes without activation of the immune response (Fitzner et al., 2011).

Exosome-mediated signaling by astrocytes seems to be involved in neurite growth and survival (Wang et al., 2011; Xin et al., 2012). Synapsin I, a vesicle protein involved in neural development, was detected in exosomes released from astrocytes and, when added to hippocampal and cortical neurons under stress conditions, exerts a protective effect via promotion of neurite outgrowth and neuronal survival, respectively (Wang et al., 2011). The transport of neuroglobin, a hypoxic neuroprotective protein, from astrocytes to neurons was also reported by an exosome-dependent mechanism (Venturini et al., 2019). The exosome-mediated transfer of prion protein, an important receptor protein that protects against oxidative stress, from astrocytes to neurons improves their protection to oxidative and ischemic stress (Guitart et al., 2016). Also, the exosome-dependent release of Hsp/c70 from astrocytes might be fundamental to locally support neighboring neurons (Taylor et al., 2007). The neuron-to-astrocyte communication is also involved in the modulation of synaptic activation. For instance, exosomes from cortical neurons modulate the activation of GLT1 protein expression in astrocytes through miR-124a found in its cargo (Morel et al., 2013).

Microglia, which constitutes the first line of defense during brain injury or infection, releases exosomes enriched in aminopeptidase CD13 involved in neuropeptide catabolism (Potolicchio et al., 2005). Exosomes released from microglia can modulate neuronal activity through the activation of sphingolipid metabolism (Antonucci et al., 2012). Serotonin stimulates the release of exosomes from microglia (Glebov et al., 2015). Moreover, neuron-to-microglia communication via exosomes enhanced the removal of degenerative neurites, thus promoting proper neuronal function and synaptic plasticity (Bahrini et al., 2015). Exosomes released from neural stem cells target microglia and act as microglial morphogens by activating transcriptional signaling pathways primarily associated with the immune system in the post-natal nervous system (Morton et al., 2018). Exosomes released from NSC seem to control the state, organization, and morphology of microglia, which in turn forms a negative feedback loop to NSC, by interfering in cell proliferation.

Exosomes are also released by the peripheral nervous system, namely, Schwann cells, which were demonstrated to further support local axonal maintenance and regeneration (Lopez-Verrilli et al., 2013). The presence of miRNA from Schwann cells was also observed in axon terminals, in a process mediated through exosome, that has an impact on gene expression and neurite growth (Chang et al., 2013b; Ching et al., 2018).

## Critical Roles of Exosomes in Neurological Disorders

EVs have been a recent object of research interest, resulting until now in several studies that search for connections between their physiological and/or pathological role and the development of neurodegenerative diseases. Several neurodegenerative disorders share other mechanistic features besides the accumulation of insoluble proteins. Exosomes are known to participate in these processes. Finding how the structure, biogenesis, cargo, communication, and final target of exosomes are altered in disease will give insights into their role in disease modulation and importantly enlighten their potential as a source of disease biomarkers. In this section, we describe the latest advances in exosome research in neurodegenerative disorders, as summarized in Table 1. Given the need for biomarkers of a large array of neurological diseases, understanding the mechanism underlying exosome regulation is of great interest for future research.

**TABLE 1:** Candidate biomarkers for neurodegenerative disorders transported in exosomes from different human biofluids.

**Exosome source**	**Huntington’s disease**	**Alzheimer’s disease**	**Parkinson’s disease**	**Amyotrophic lateral sclerosis**
*“Non-specific”* exosomes	Absence of mHTT (Denis et al., 2018)	Phosphorylated tau (Saman et al., 2012) ↓REST (Goetzl et al., 2015)	↑miR195 and miR24 ↓miR19b (Cao et al., 2017)	Altered levels of miRNA (Yelick et al., 2020)
NDEs		↑Aβ1-42 ↓Synaptic proteins ↑TDP43 (Fiandaca et al., 2015; Goetzl et al., 2016, 2018; Zhang et al., 2020) ↓synaptophysin, synaptotagmin and SNAP-25 (Goetzl et al., 2016; Agliardi et al., 2019) ↓miR132 and miR-212 (Walsh et al., 2019)	↑DJ1 and α-synuclein (Zhao et al., 2019) ↑α-synuclein and clusterin (Jiang et al., 2020) ↑α-synuclein (Shi et al., 2014; Jiang et al., 2020; Fu et al., 2020; Niu et al., 2020)	Altered levels of miRNA (Katsu et al., 2019; Banack et al., 2020)

**NDEs, neuronally derived exosomes; mHTT, mutant huntingtin; REST, repressor element 1-silencing transcription factor; Aβ1-42, amyloid beta-42; TDP43, TAR DNA-binding protein 43; SNAP-25, 25 kD synaptosomal protein; miR, micro RNA.**

### Huntington’s Disease

The pathology of HD is caused by a CAG expansion in the *HTT* gene (Huntington’s Disease Collaborative Research Group, 1993), which encodes a protein with a polyglutamine expansion at the N-terminal, mutant huntingtin (mHTT). Accumulation of the mutated protein results in cognitive deficits and involuntary movements, correlated with a selective loss of striatal medium spiny neurons and cortical atrophy (Roos, 2010). The implication of the possible transfer of pathological biomolecules through a non-cell-autonomous pathway in HD has aroused attention after Cicchetti and colleagues showed that healthy fetal neural tissue engrafted in HD patients displayed mHTT aggregates several years after transplantation (Cicchetti et al., 2014). The unconventional mHTT spread throughout exosome transport was then suggested as a novel mechanism involved in HD pathology and opened new possibilities to find therapeutic targets aiming to mitigate this neurodegenerative condition.

Several studies suggested that exosomes can transport the expanded polyglutamine tract of HTT RNA and protein, as well as mHTT aggregates (Jeon et al., 2016; Zhang et al., 2016). Zhang and colleagues showed that after infecting human HEK293T cells with a lentivirus encoding mutant exon 1 huntingtin fragments, these cells secreted exosomes containing transcripts of the mutant polyglutamine tail and HTT peptides (Zhang et al., 2016). The addition of these exosomes to mouse striatal cells carrying both normal and mutant CAG repeats (*Q*^7/7^, *Q*^111/111^, respectively) led to the uptake of RNA but curiously not the protein (Zhang et al., 2016). However, others have acknowledged the uptake of exosomes containing mHTT by SH-SY5Y cells when exposed to conditioned media from HEK293 cells overexpressing GFP-mHtt-Q19 and -Q103 (Jeon et al., 2016). This is a topic with variable outcomes since the presence of mHTT is not always observed in exosomes, as described for astrocytes of an HD knock-in mouse model (*HD140Q KI*) (Hong et al., 2017). Huntingtin is a large protein with 350 kDa, which makes it difficult to be fully packed into exosomes. mHTT spreading through the exosome pathway is a complex and not yet defined mechanism that surely has an associated variability between HD models. However, strong evidence supports the idea that mHTT can be carried by exosomes reinforcing the need to better clarify how the protein is loaded into exosomes. The acknowledgment that mHTT is present in exosomes was shown when murine embryonic fibroblasts (MEFs) overexpressing the exon 1 of the *Htt* gene showed constitutive interaction of mHTT with exosome structural proteins Alix and TSG101 mediated by Transglutaminase 2 (Diaz-Hidalgo et al., 2016). In HD astrocytes, mHTT was shown to impair the exosome secretion by decreasing αB-crystallin levels, a glial protein involved in exosome secretion. The overexpression of αB-crystallin could revert the decrease in exosome release and simultaneously decreased the mHTT aggregates in the striatum of HD140Q KI mice (Hong et al., 2017). On the other hand, in the same study, the injection of exosomes from WT astrocytes was described to prevent aggregation of mHTT (Hong et al., 2017). The exosome transport of mHTT was confirmed when neurons differentiated from WT mice NSC displayed mHTT aggregates upon co-culture with HD fibroblasts (143 CAG repeats); moreover, mice injected with HD-derived exosomes also revealed that mHTT aggregates specifically in the DARPP-32 + medium spiny neurons of the host’s striatum. Eight weeks post-incubation, the animals exhibited motor impairment and cognitive deficits (Jeon et al., 2016). In contrast, exosomes isolated from platelets of HD patients failed to show any presence of mHTT (Table 1; Denis et al., 2018). Altogether, this data awareness on the importance of exosomes in the progression and pathophysiology of HD supports the need for deeper research in exosome-mediated mechanisms.

### Alzheimer’s Disease

Alzheimer’s disease (AD), the most common form of dementia, has, as a causative mechanism, the accumulation of aggregated β-amyloid (Aβ) in the brain and phosphorylated tau in neurofibrillary tangles (Palmqvist et al., 2017). The accumulation of Aβ fibrils can precede symptoms for decades, which has long intrigued researchers looking for the mechanisms by which neurodegeneration occurs (Sperling et al., 2014). The discovery of multivesicular bodies (MVBs) as a site for the accumulation of Aβ42 in pre- and postsynaptic compartments preceded the later acknowledgment that the cleavage of β-amyloid peptides occurs in early endosomes before routing to MVB and the resulting products released from cells through exosomes (Takahashi et al., 2002; Rajendran et al., 2006). The presence of Alix associated with amyloid plaques in post-mortem AD patient’s brains, a fact not observed in healthy brains, reinforced the existence of a role for Aβ-associated exosomes in amyloid plaque formation (Rajendran et al., 2006). The presence of secretases (γ-, α-, and β-secretases), enzymes involved in the proteolytic cleavage of APP, in isolated exosomes from an AD animal model raised the possibility for these vesicles to be a potential site of APP cleavage (Sharples et al., 2008). Indeed, it was observed that APP goes through cleavage inside exosomes, with existing evidence that products resulting from APP processing are present in exosomes from AD human and mouse AD model brains, evidencing the exosome intervention in the pathological process (Vingtdeux et al., 2007; Sharples et al., 2008; Perez-Gonzalez et al., 2012; Guix et al., 2017; Laulagnier et al., 2018).

After evidence of existing Aβ in association with exosomes, the concern was to understand whether these vesicles contribute, as a vehicle, to spreading the pathology or can be protective by clearing the toxic elements associated with AD pathophysiology. Exosomes derived from neuroblastoma cells injected into the brain of the hAPP-J20 AD mouse model were able to trap Aβ bound to the glycosphingolipid surface and later transport to microglia for degradation. These findings supporting the exosome mentioned “clearance role” through the decrease in the levels of Aβ and Aβ-mediated synaptotoxicity (Yuyama et al., 2014). Altogether, these data reinforce the capacity of exosomes to carry Aβ and “deliver” their cargo to microglia, reducing the levels of synaptic toxicity and inflammatory response.

The levels of certain enzymes and structural components in exosomes promote Aβ elimination by microglial and lysosomal processes, including the insulin-degrading enzyme, ceramide, or the ganglioside GM1 (Tamboli et al., 2010; Wang et al., 2012; Yuyama et al., 2012).

Thereby, there is evidence that microglial phagocytic action on Aβ is promoted by exosomes being mediated by sphingolipids integrating their structure. Moreover, β-amyloid oligomers (Aβo) incubated with exosomes were sequestered to their superficial structure by interacting with surface proteins including the prion protein (PrP^C^) (An et al., 2013). The sequestration of Aβo by exogenous exosomes can exert a protective effect against the synaptic disruption induced by Aβ (An et al., 2013). Indeed, PrP^C^ plays a dual role: a protective one, by capturing Aβ in exosomes, through superficial recognition, thus facilitating the formation of amyloidogenic fibrils and preventing the derivative neurotoxic complications mediated by Aβo and a neurotoxic effect when expressed in the neuronal surface as a receptor for Aβ (Falker et al., 2016). Other exosome components from the outer membrane were reported to be associated with Aβ, including glycans of glycosphingolipids and GM1 ganglioside, which was seen to induce exosome release and promote Aβ fibril formation (Yuyama et al., 2008, 2014). Hypoxia can accelerate the production of Aβ by increasing the activity of β and PS1/γ-secretase. In SH-SY5Y cells (expressing human WT APP695), maintained in hypoxic conditions, was observed an increase in Aβ40 and Aβ42 content in exosomes (Xie et al., 2018).

Although the abovementioned studies reveal an eliminatory role for exosomes to discard Aβ, C-terminal fragments, and full-length APP, recent data suggest that these vesicles can also transport Aβo, further contributing to the neurotoxicity seen in AD. The use of biological material/samples from AD patients can more accurately help to unravel what specifically happens in the development of the disease other than cellular or animal models. Recently, a study in post-mortem tissue from the temporal neocortex of AD patients showed that flotilin-1 and Aβo co-labeled and there were increased levels of oligomers in exosomes compared to controls (Sinha et al., 2018). Incubation of human-derived induced-pluripotent stem (hiPSC)-differentiated neurons and SH-SY5Y cells with labeled exosomes isolated from brain tissue of AD patients revealed the incorporation of vesicles carrying Aβo, leading to subsequent cytotoxicity when comparing to healthy brains (Sinha et al., 2018). The ablation of Aβo spreading was achieved by using siRNA to inhibit transcription of TSG101 and VPS4A, proteins involved in exosome formation and secretion (Sinha et al., 2018). In sum, these studies confirmed the transport of Aβo, APP, and its cleaved products in exosomes and their further uptake and internalization by neuronal cells. Moreover, the neurotoxic effects of adding exosomes derived from AD human plasma, cell, and animal models or previously exposed to toxicity-inducing Aβ-protofibrils has also been described (Eitan et al., 2016; Beretta et al., 2020).

Tau has also been found in exosomes isolated from CSF and brain of AD patients (Saman et al., 2012; Wang et al., 2017; Guix et al., 2018; Crotti et al., 2019; Ruan et al., 2021). In accordance, the injection into WT mice of exosomes from iPSC-derived neurons and post-mortem brain of AD patients led to the increase in *tau* phosphorylation and formation of inclusions (Aulston et al., 2019; Ruan et al., 2021).

Generally, the diagnosis of AD patients is based on the expression of tau and Aβ; however, the observation that exosomes from AD patients have differences in cargo has sparked curiosity in the field of biomarkers (Table 1). In accordance, exosomes isolated from plasma of AD patients exhibited decreased levels of transcription factors involved in neuronal protection from external damages like the repressor element 1-silencing transcription factor (REST), as already observed in individuals at pre-clinical stages without cognitive impairments (Goetzl et al., 2015).

The recent developments in the methods to isolate exosomes of neuronal origin from plasma have gained interest as they may include more sensitive and accurate neuronal biomarkers than total plasma exosomes (Table 1). Some reports have shown the presence of higher levels of Aβ_1__–__4__2_ and TAR DNA-binding protein 43 (TDP43) and decreased levels of synaptic proteins, as synaptophysin, synaptotagmin, and 25-kDa synaptosomal-associated protein (SNAP-25), in NDEs from AD patients (Fiandaca et al., 2015; Goetzl et al., 2016, 2018). Importantly, these first two presynaptic proteins, in particular, were already diminished at the pre-clinical stage and were correlated with the cognitive decline, as evaluated by the mini-mental state examination (Goetzl et al., 2016). In accordance, the exosome-associated growth-associated protein (GAP43) neurogranin, SNAP25, and synaptotagmin 1 proteins can be detected in AD up to several years before clinical onset (Jia et al., 2020). Following the same procedure, Zhao and colleagues showed that NDE Aβ_1__–__42_ levels allow discriminating between cognitive controls, mild cognitive impairment (MCI) patients, and AD dementia patients and predicting the risk of MCI progressing to AD dementia (Zhao et al., 2020). The levels of miR132 and miR-212 were also shown to be decreased in NDEs from AD patients (Walsh et al., 2019). Another comprehensive study involving EV isolated from post-mortem brain tissue from AD subjects has provided more insights into differential RNA biotype exosome content, namely, expression patterns of AD-associated miRNA compared with brain homogenates, adding to the pallet of possible targets for studying AD (Cheng et al., 2020). The NDEs’ novel approach, considered a liquid biopsy of the brain, resorts to exosomes released from neuronal cells found in the peripheral source, to discover more accurate biomarkers for neurodegenerative disorders. This technique has mainly been used in AD studies; however, strong proof of concept has also been assessed in other neurodegenerative diseases.

### Parkinson’s Disease

Parkinson’s disease (PD) is the most common neurodegenerative movement disorder affecting 0.3% of the general population. The pathological hallmark is the loss of dopaminergic neurons in the substantia nigra *pars compacta* (SNpc) of the *striatum* and the accumulation of α-synuclein in cytoplasmic inclusions called Lewy Bodies (Balestrino and Schapira, 2020). The formation of Lewy Bodies in healthy transplanted areas of PD patients years after grafting raised curiosity for the study of possible non-autonomous spreading mechanisms including related EVs (Kordower et al., 2008).

The first evidence of exosome involvement in PD was the presence of α-synuclein in Alix- and flotillin-positive vesicles derived from α-synuclein-expressing cells (Emmanouilidou et al., 2010). In 2012, the oligomeric form of α-synuclein was shown to be transported via exosomes, and additionally that exosomes may favor the formation of α-synuclein aggregates due to its lipid and/or protein composition thus facilitating the uptake of α-synuclein by cells (Danzer et al., 2012; Grey et al., 2015; Delenclos et al., 2017). Moreover, oligomerized α-synuclein seemed to be preferentially uptaken by cells when associated with exosomes rather than isolated, reinforcing the role of exosomes to transfer and deliver toxic oligomeric proteins to cells (Delenclos et al., 2017). Similar results were obtained when exosomes obtained from CSF of PD patients were added to H4 glioma cells, resulting in the elevated formation of α-synuclein oligomers (Stuendl et al., 2016). Surprisingly, this study reported reduced levels of α-synuclein in exosomes from CSF of early stage PD patients when compared to controls. On the other hand, others claim that the presence of α-synuclein seemed to be elevated in exosomes from plasma of PD patients, suggesting a positive correlation between exosome-associated α-synuclein levels and disease severity studies (Shi et al., 2014). In fact, the presence of α-synuclein in L1CAM-positive NDEs validated the protein as a biomarker for PD with origins in the nervous system (Shi et al., 2014). Henceforward, investigators have searched for mechanisms behind the release of exosomes aiming to manipulate it and find new outcomes to treat neurodegeneration in PD. α-synuclein was also shown to induce the release of exosomes by microglia cells in mice, thus promoting apoptosis, as well as lysosomal dysfunction in α-synuclein overexpressing SH-SY5Y cells, leading to an increase in the release of exosome-associated α-synuclein (Alvarez-Erviti et al., 2011a; Chang et al., 2013a). Additionally, when exosomes from serum of PD patients, highly enriched in α-synuclein, were injected in mice, it induced protein aggregation along with a neuroinflammatory response, reinforcing the neurotoxic role of exosome transfer in PD (Han et al., 2019). Alterations in genes’ expression like ATPase cation transporting 13A2 (ATP13A2) and β-glucocerebrosidase have also been associated with differences in the exosome-associated release of α-synuclein (Kong et al., 2014; Papadopoulos et al., 2018). While the overexpression of ATP13A2 (overexpressed in neurons of PD patients) in SH-SY5Y differentiated cells caused a decrease in intracellular α-synuclein with enhanced levels in exosomes, inhibition of β-glucocerebrosidase enzymatic activity *in vivo* caused the enhancement of α-synuclein release in association with exosomes and the levels of α-synuclein oligomers (Kong et al., 2014; Papadopoulos et al., 2018).

Altogether, these data suggest a transmission role for exosomes, as carriers of pathogenic/toxicity-inducing elements. The same applies to the loading of nucleic/proteic fragments that could rescue neurotoxic processes happening in PD contexts. Strikingly, a study showed that PD plasma-derived exosomes, when added to rat cortical neurons, were capable of diminishing apoptosis and increase metabolic activity when compared to the addition of exosomes from healthy individuals (Tomlinson et al., 2015). A possible explanation for the different outcomes of this study could be the incapacity to detect α-synuclein in PD-derived serum microvesicles. In line with these results, another study showed that a mouse model of PD equally benefited from injection with exosomes from blood of healthy controls, by experiencing diminished cell loss and movement alterations, demonstrating the therapeutic role of exosomes as potential rescuers in neurodegeneration (Sun et al., 2020).

Access to plasma samples from PD patients also allowed the isolation of plasma NDEs and the detection of more sensitive disease biomarkers, such as DJ1 and α-synuclein (Table 1; Zhao et al., 2019). The same increased levels of α-synuclein were, however, not confirmed in exosomes isolated from the urine of PD patients (Ho et al., 2014). More recently, in plasma L1CAM-positive vesicles, an increase of α-synuclein levels in combination with clusterin, a stress-induced chaperone, allowed the differentiation of patients with PD from those with atypical parkinsonism (Jiang et al., 2020). The α-synuclein levels in NDEs from plasma and serum of PD patients were shown to be augmented but with some divergences between studies on how this aspect correlates with disease progression (Fu et al., 2020). The number of NDEs was described to be higher in plasma of PD patients and correlated with either early or very advanced stages of the disease (Ohmichi et al., 2019). NDEs positive for L1CAM were also used as a tool to assess the concentration of proteins of interest in the serum of PD patients who participated in a clinical trial for the study of possible beneficial effects of a diabetes type 2 treatment drug (Athauda et al., 2019). The study of the mechanisms linking PD and exosome is of major interest to understand the neurotoxic events in PD contexts and brings hope for new therapeutic approaches focusing on nanovesicles as key interlocutors.

### Amyotrophic Lateral Sclerosis

Amyotrophic lateral sclerosis (ALS) affects both upper motor neurons and lower motor neurons leading to motor and extra-motor symptoms. The majority of cases is related to alterations in four genes, namely, C9orf72, TARDBP (encoding TAR DNA-binding protein 43, TDP43), SOD1 (encoding superoxide dismutase), and FUS (encoding RNA binding protein FUS), which results in aggregation and accumulation of ubiquitinated protein inclusions in motor neurons (Mejzini et al., 2019).

ALS was first associated with exosome trafficking in 2007 when a study showed the transport of both WT and mutant Cu/Zn superoxide dismutase (SOD1) by exosomes in a cell model for ALS (mouse neuron-like NSC-34 overexpressing human mutant SOD1) (Gomes et al., 2007). Further ahead, Grad and colleagues demonstrated that the same ALS cellular model was capable of incorporating misfolded SOD1, pointing that this protein was localized in the external part of exosomes isolated from the supernatant of cells transfected with human WT and mutant GFP-SOD1 (Grad et al., 2014). Astrocytes from primary cultures overexpressing mutant SOD1 released more exosomes than controls and more efficiently transferred these proteins, resulting in the loss of motor neurons as shown by the decrease in the SMI32/NeuN ratio in cultures where exosomes were added (Basso et al., 2013). Indeed, the release of mutant SOD1 by EV has been observed in both astrocytes and neurons from an ALS animal model (transgenic SOD1^G93A^ mouse model) (Basso et al., 2013; Silverman et al., 2019). Moreover, mutant SOD1 was also present in EV from the brain and spinal cord of ALS patients, evidencing the spreading role of these vesicles (Silverman et al., 2019). In accordance, an EV-associated miRNA (miR-494-3p) released by astrocytes obtained through direct conversion of fibroblasts of ALS patients contributed to the loss of motor neurons in mice (Varcianna et al., 2019). Additionally, alteration of miR-124 levels associated with inflammatory processes was observed in exosomes from NSC-34 (motor neuron-like cultures) transfected with mutant SOD1 (Pinto et al., 2017). These exosomes were preferentially incorporated by microglial cells (co-culture of microglia and NSC) and lead to the decrease of microglia phagocytic ability.

The continuous search for the role of exosomes in ALS motivated concerns about the plausible spreading of other pathological agents in the disease than the mutant protein mainly implied, SOD1. Further analysis showed that TDP-43 protein was transported through exosomes isolated from the CSF of ALS patients (also suffering from frontotemporal dementia) to U251 human glioma cells (Ding et al., 2015). The uptake of these exosomes was accompanied by an increase in apoptosis- and autophagy-related proteins, which demonstrated that the transport of toxic cargo by nanovesicles can spread and promote negative effects throughout cells.

The identification of miRNA (e.g., miR-124-3p, hsa-miR-4736, and miR-146a-5p) with altered levels in both neuronal-derived and total exosomes from ALS patients (CSF and plasma) gave some insight into novel targets to overcome the pathology (Table 1; Banack et al., 2020). Nonetheless, the research regarding the role of exosome vesicles in ALS has still a long way to go to allow a better understanding of how we can use the already acquired knowledge to mitigate the progression of these neurodegenerative processes.

## Future Perspectives: Diagnostic and Therapeutic Potentials of Exosomes

In the last decade, the research into the exosome’s biological characteristics has seen exponential growth. Exosomes are secreted from virtually all types of cells acting as mediators of intercellular communication physiologically. Interestingly, evidence has been reported that exosomes are involved in the development of human neural circuits by regulating signaling pathways. The maturation and function of neurogenic niches are highly regulated by miRNA (e.g., miR-let7b and miR-9 regulates NSC proliferation and differentiation; miR-34a promotes NSC differentiation and neuron maturation among others) and most of them were found in exosome cargo from different types of cells (Bátiz et al., 2015). Moreover, exosomes have shown the potential to restore brain cellular function in the NSC model of Rett syndrome incubated with isogenic control iPSC-derived exosomes, resulting in amelioration of the developmental deficit associated with the disease (Sharma et al., 2019). Exosomes from NSCs were also able to reduce the lesion extension in a spinal cord injury mouse model, improve functional recovery, and mitigate neuroinflammation by inducing autophagy (Rong et al., 2019). These data are feasible such that exosome content can target NSC/NPC to modulate the neurogenic process and influence neuronal plasticity. Importantly, exosomes from NSCs exert anti-inflammatory, neurogenic, and neurotrophic effects that are likely to be useful to treat conditions such as dementia, AD, HD, PD, ALS, stroke, traumatic brain injury, multiple sclerosis, and major depressive disorder.

In contrast, exosomes have been suggested to be important players in the propagation of neurodegenerative diseases by providing a mechanism for the spreading of toxic content such as misfolded, aggregated forms of proteins. In the last few years, a causal connection between exosomes and the development and progression of CNS diseases such as AD, PD, prion disease, multiple sclerosis, traumatic encephalopathy, and stroke, among others, has been explored (Liu et al., 2019). These diseases have in common the activation of the autophagic–lysosomal pathways for the elimination of misfolded proteins. Exosomes can act as an alternative pathway to the overload of these protective cellular routes by loading and secreting the deleterious proteins (ExoCarta), although the mechanism is not yet fully comprehended. Our understanding of the cellular and molecular mechanisms involved in the biogenesis, release, uptake, and function of EV remains elusive, and further studies are needed to help clarify these aspects. Whereas the importance of exosomes in cell communication is undisputed, the knowledge of how they can influence and regulate cell responses is critical to deciphering their potential physiological and pathological impacts. While the current studies offer contradictory data on the favorable or adverse effects exerted by exosomes, they are not certainly bystanders in any step of these biological processes.

A major pitfall in the exosome field is the technical difficulties in the isolation and characterization of “pure” exosomes. The exosome cargo is enriched in nucleic acids, proteins, lipids, amino acids, and metabolites that vary according to the cell of origin. The heterogeneity among preparations and the possible contamination of EVs/exosome fraction with body fluid contaminants might interfere with the characterization of vesicular cargo. Our knowledge about exosome research is challenged due to the lack of reliable and accurate isolation protocols, the lack of selective biomarkers, and the lack of high-resolution characterization techniques. Routine isolation methods include ultracentrifugation, density-gradient centrifugation, and ultrafiltration with all having their own advantages and disadvantages, and a universally accepted methodology for exosome isolation is still an intense debate. Identifying and separating different subpopulations of exosomes from specific cellular types will provide tremendous advantages in defining an exosome biosignature to act as a diagnostic biomarker. Isolation of neural-derived exosomes from human plasma is a particularly attractive method to improve the specificity of disease fingerprints. The immunoprecipitation of neuronal exosomes by antibodies targeting the marker proteins NCAM or L1CAM has proven to significantly increase the accuracy of exosome content as a disease biomarker. NCAM and L1CAM are not exclusive from neurons, despite their enrichment, impelling the search for more precise markers that will allow to obtain purer exosome populations from different brain cell types and eventually compare exosome biomarkers in the same sample. Importantly, there is an urge for more robust biomarkers obtained through a minimally invasive method for diagnostic purposes, preferably in the early stages of the disease, to monitor disease progression and to evaluate treatment response.

Exosomes are a promising tool with potential clinical application both as a diagnostic biomarker and as a therapeutic agent, particularly as drug/gene delivery systems. For a long time, mesenchymal stem cell-derived exosomes have been used in a myriad of pathological conditions with success (Xin et al., 2012; Ibrahim et al., 2014; Hao et al., 2017; Mendt et al., 2019). Undifferentiated iPSCs were also reported to release an elevated number of exosomes that exert multiple protective effects in different situations such as in cardiovascular diseases or by reducing senescence and stimulating proliferation of human dermal fibroblasts (Wang et al., 2015; Oh et al., 2018). Exosomes derived from human iPSC-neural stem cells led to significant improvements at the tissue and functional levels in different animal models of stroke, strongly supporting exosomes as a biological treatment (Webb et al., 2018a,b). These studies emphasize the important role of exosomes derived from neural cells in maintaining neural functions.

Exosomes have natural cargo transportation properties, turning them into an excellent vehicle for transferring bioactive molecules such as drugs, proteins, non-coding miRNA, and gene therapeutic agents to specific cells involved in CNS diseases. Exosomes combine a myriad of advantages as nanocarriers including stability, biocompatibility, low immunogenicity, ability to cross the blood–brain barrier, and efficient cellular uptake that can be modulated to target specific cells according to appropriate membrane proteins. The field of nanotherapy centered on nanoparticle-based exosome mimetics has evolved rapidly and can be a promising tool for drug delivery. Of major interest is the incorporation of miRNA into exosomes to be delivered into recipient cells for protective effects. However, despite the advances stressed, there are many challenging points to consider. One is related to the low yield of exosomes obtained from cells since large-scale production of exosomes for clinical use must be achieved. Furthermore, the use of exosomes for therapeutic purposes should clarify the dose–response output and the distribution and most importantly increase the specificity for the target cells. The lack of an efficient method for cargo loading into the exosomes is also a limitation. The current strategies include conjugation of harvested exosomes with drugs by different methods (e.g., electroporation, sonication, extrusion, and freeze/thaw), incubating cells with compounds to be incorporated into the exosomes, or transfection of donor cells with active molecules before the collection of the exosomes. So far, none of the strategies have been validated for clinical use. The short run is expected to have exosome-based therapeutics treating a wide range of maladies. Meanwhile, fully exploiting the exosomes’ potential is critical to define the safest and more efficient modes of delivery into the target tissue, either by systemic administration or by local delivery. Exosomes in circulation are rapidly sequestered by organ or cleared, remaining in the serum less than 5% at 5 min upon intravenous injection (Takahashi et al., 2013).

Importantly, the examples discussed above have demonstrated that exosomes comprise a great potential for the diagnosis and treatment of neurodegenerative disorders. Currently, the efforts in the field are focused on not only obtaining exosomes from specific brain cell types but also exploring the possibility of targeting exosomes to a specific neuronal type in the brain. These findings are expected to open up new avenues for pharmacological treatments with minor side effects. Overall, we have come a long way in exosome research, although several obstacles remain to be overcome before transitioning from innovation to widespread clinical use.

## Author Contributions

MB, RV, and CL conceived, wrote, and participated in the revising of the manuscript. All authors have approved the final version of the manuscript.

## Conflict of Interest

The authors declare that the research was conducted in the absence of any commercial or financial relationships that could be construed as a potential conflict of interest.
